# A versatile model for lifetime of a component under stress

**DOI:** 10.1038/s41598-023-47313-3

**Published:** 2023-11-18

**Authors:** Fatimah E. Almuhayfith, Talha Arslan, Hassan S. Bakouch, Aminh M. Alnaghmosh

**Affiliations:** 1 Department of Mathematics and Statistics, College of Science, King Faisal University, Alahsa, 31982 Saudi Arabia; 2 Department of Econometrics, Van Yüzüncü Yıl University, Van, Turkey; 3 Department of Mathematics, College of Science, Qassim University, Buraydah, 51452 Saudi Arabia; 4 Department of Mathematics, Faculty of Science, Tanta University, Tanta, 31111 Egypt

**Keywords:** Applied mathematics, Statistics

## Abstract

In this study, a versatile model, called $\alpha$-monotone inverse Weibull distribution ($\alpha$IW), for lifetime of a component under stress is introduced by using the $\alpha$-monotone concept. The $\alpha$IW distribution is also expressed as a scale-mixture between the inverse Weibull distribution and uniform distribution on (0, 1). The $\alpha$IW distribution includes $\alpha$-monotone inverse exponential and $\alpha$-monotone inverse Rayleigh distributions as submodels and converenges to the inverse Weibull, inverse exponential, and inverse Rayleigh distributions as limiting cases. Also, slash Weibull, slash Rayleigh, and slash exponential distribuitons can be obtained under certain variable transformation and parameter settings. The $\alpha$IW distribution is characterized by its hazard rate function and characterizing conditions are provided as well. Maximum likelihood, maximum product of spacing, and least squares methods are used to estimate distribution parameters. A Monte-Carlo simulation study is conducted to compare the efficiencies of the considered estimation methods. In the application part, two practical data sets, Kevlar 49/epoxy and Kevlar 373/epoxy, are modeled via the $\alpha$IW distribution. Modeling performance of the $\alpha$IW distribution is compared with its rivals by means of some well-known goodness-of-fit statistics and results show that $\alpha$IW distribution performs better modeling than them. Results of comparison also indicate that obtaining the $\alpha$IW distribution by using the $\alpha$-monotone concept is cost effective since the new shape parameter added to the distribution by using the $\alpha$-monotone concept significantly increases the modeling capability of the IW distribution. As a result of this study, it is shown that the $\alpha$IW distribution can be an alternative to the well-known and recently-introduced distributions for modeling purposes.

## Introduction

Inverse Weibull (IW) distribution, also known as Fréchet or type-II extreme value distribution, is introduced by Keller and Kanath^1^ to model the degeneration phenomena of mechanical components, such as dynamic components (piston, crankshaft, etc.) of diesel engines; see also Nelson^2^ in the context of modeling breakdown of insulating fluid. Later on, it has been used in analysing data from different areas of science, e.g. actuaria, agricaulture, energy, hydrology, medicine, and so on.

The probability density function (pdf) of the IW distribution is

1 $$\begin{aligned} f_{X}(x; \beta , \sigma )=\beta \sigma x^{-(\beta +1)}\exp \big ({-\sigma x^{-\beta }}\big ); \quad x>0, \quad \beta , \sigma >0, \end{aligned}$$

and its cumulative distribution function (cdf) is

2 $$\begin{aligned} F_{X}(x; \beta , \sigma )=\exp \big ({-\sigma x^{-\beta }}\big ); \quad x>0, \quad \beta , \sigma >0, \end{aligned}$$

where $\beta$ and $\sigma$ are the shape and scale parameters, respectively. Herein after, random variable having pdf in (1) is denoted by $X \sim IW(\beta , \sigma )$. As stated in Helu^3^, the IW distribution has longer right tail than the other well-known distributions and also has hazard function like log-normal and inverse Gaussian distributions.

The IW distribution and its sub-models inverse Rayleigh and inverse exponential distributions are widely used in reliability engineeering. However, in some cases, IW and its sub-models can not model reliability data adequately. Therefore, generalized/extended versions of the IW distribution are proposed to improve its modeling capability, i.e. to model reliability data more accurately; see for example Chakrabarty and Chowdhury^4^, Fayomi^5^, Hanagal and Bhalerao^6^, Jahanshahi et al.^7^, Saboori et al.^8^, Afify et al.^9^, Hussein et al.^10^ and references given them. Note that IW distribution is also called Fréchet distribution. Therefore, we recommend reading Afify et al.^11^, Hussein et al.^10^ and references given them in the context of extension/generalization of the Fréchet distribution.

There are quite a variety of methods for extending/generating distribution; see Lee et al.^12^ for an overview on it. In this context, slash distribution introduced by Andrews et al.^13^ has been popular. Later on, various slash distributions were introduced; e.g., Oliveres-Pacheco et al.^14^, Iriarte et al.^15^, Korkmaz^16^, Gómez et al.^17^, and references that given by them for univariate slash distributions.

Recently, Jones^18^ considered the distribution of $X \times Y^{\frac{1}{\alpha }}$ that is formally different form of the slash distribution, and called it as $\alpha$-monotone density. Here, *X* and *Y* are independent random variables following distributions on $\mathbb {R^{+}}$ and uniform distribution having range 0 to 1, i.e., U(0,1), respectively. See Jones^18^ and Arslan^19–21^ for the theoretical view points of the $\alpha$-monotone distribution and application of it, respectively. Arslan^20,21^ show that the $\alpha$-monotone concept is easly applied for the baseline distribution and make significant effect on the modeling capability of the baseline distribution.

It is known that IW distribution is a well-known candidate in modeling lifetime data. However, its modeling performance may be inadequate since it has one shape parameter; therefore, it has to be improved in such cases. For example, when lifetime of a component under the stress wanted to be modeled and the effect of stress level wanted to be estimated, a new parameter, called stress parameter or shape parameter, may be included into density of the IW distribution. As a consequance, lifetime of a component under the stress is expected to be lower than the routine case. The division operation is usually used to reduce the value of a random variable arbitrarily. However, multiply it by a random variable taking value between 0 and 1 can also be used. Here, the reason prefering the multiplication rather than division is because having a very useful variable following U(0,1). Therefore, a random variable *T* defined as $T=X \times Y^{\frac{1}{\alpha }}$ is very important in lifetime analysis. The *T* can be represent the lifetime of *X* under the stress.

Since the inherent of $\alpha$-monotone distribution, a random variable having an $\alpha$-monotone distribution will preciously represent a lifetime of a component under the stress. In this regard, $\alpha$-monotone distribution can be an alternative to the IW distribution. However, to the best of authors' knowledge, there are limited number of studies in the literature concerning the $\alpha$-monotone distribution spesifically in modeling the lifetime data. The motivation of this study comes from filling the gap in the literature about the $\alpha$-monotone distributions in lifetime data modeling. Therefore, in this study, a practical model, for lifetime of a component under stress, is proposed. The pdf of the proposed model meets the condition for having $\alpha$-monotone density; therefore it is called $\alpha$-monotone IW ($\alpha$IW) distribution. Also, the cdf and hazard rate function (hrf) of the $\alpha$IW distribution are obtained; then, the $\alpha$IW distribution is characterized by its hrf and characterizing conditions are provided as well. The *r*-th moment of the $\alpha$IW distribution is also formulated. Furthermore, it is showed that the $\alpha$IW distribution can be expressed as a scale-mixture between the IW and U(0, 1) distributions. Data generation process is also developed by using the stochastic representation of the random variable having the $\alpha$IW distribution. Maximum likelihood, maximum product of spacing, and least squares estimation methods are used to estimate parameters of the $\alpha$IW distribution. The $\alpha$IW distribution includes the $\alpha$-monotone inverse Rayleigh, $\alpha$-monotone inverse exponential, IW, inverse Rayleigh, and inverse exponential distributions for the different parameter settings and limiting cases. Thus, it can be considered as a general class of the IW distribution by adding a new shape parameter that allows to distribution being more flexible than the IW distribution. In light of this, the $\alpha$IW can be an alternative to the IW and its rivals in lifetime data analysis.

The rest of the paper is organized as follows. The $\alpha$IW distribution, its characterization, and properties are given; then submodels of the $\alpha$IW distribution are obtained and data generation process for the $\alpha$IW distribution is provided. By the following sections, parameter estimation for the $\alpha$IW distribution is handled and real-life data sets are used to show modeling capability of the $\alpha$IW distribution and compare it with its rivals. The paper is finalized with some concludings and remarks.

## The $\alpha$IW distribution

### Proposition 1

A random variable *T* defined by $T=X \times Y^{1/\alpha }$, where X $\sim$ IW($\beta , \sigma$) and Y $\sim$ U(0, 1) are independent random variables, follows the $\alpha$IW distribution having the pdf

3 $$\begin{aligned} f_{T}(t;\alpha , \beta , \sigma )=\frac{\alpha ^2}{\beta \sigma ^{\alpha /\beta }} \Gamma \left( \dfrac{\alpha }{\beta }\right) t^{\alpha -1} G\left( t^{-\beta }; \dfrac{\alpha }{\beta }+1, \sigma \right) ; \quad t>0, \quad \alpha , \beta , \sigma >0. \end{aligned}$$

Here, $\Gamma (a)=\displaystyle \int _{0}^{\infty }u^{a-1}e^{-u}du$ and $G(t;a,b)=\frac{a^b}{\Gamma (a)}\displaystyle \int _{0}^{z}u^{a-1} \exp (-b u) du$ are the gamma function and the cdf of the gamma distribution, respectively.

### Proof

The proof is completed by using the Jacobian transformation, where *J* is the Jacobian,

$$\begin{aligned} \left. \begin{array}{cc} T= &{} \displaystyle \frac{X}{Y^{-\frac{1}{\alpha }}} \\ &{}\\ W= &{} Y\\ \end{array} \right\} \Rightarrow \left. \begin{array}{cc} X= &{}T W^{-\frac{1}{\alpha }}\\ Y= &{} W \\ \end{array} \right\} \Rightarrow J =\left| \begin{array}{cc} \displaystyle \frac{\partial X}{\partial T} &{}\displaystyle \frac{\partial X}{\partial W} \\ &{} \\ \displaystyle \frac{\partial Y}{\partial T} &{} \displaystyle \frac{\partial Y}{\partial W}\\ \end{array} \right| =\left| \begin{array}{cc} w^{-\frac{1}{\alpha }} &{}-t\frac{1}{\alpha }w^{-\frac{1}{\alpha }-1} \\ 0 &{} 1 \\ \end{array} \right| = w^{-\frac{1}{\alpha }} . \end{aligned}$$

Then, the joint pdf of *T* and *W* is

$$\begin{aligned} f_{T,W}(t,w)=\beta \sigma t^{-(\beta +1)} w^{\frac{\beta }{\alpha }}\exp (-\sigma t^{-\beta } w^{\frac{\beta }{\alpha }}). \end{aligned}$$

The marginal pdf of the random variable $T$ is obtained immediately by taking integration with respect to the random variable $W$ using the transformation $t^{-\beta } w^{\frac{\beta }{\alpha }}=u$. Herein after, random variable having pdf in (3) is denoted by $T \sim \alpha \text {IW}(\alpha , \beta , \sigma )$. $\square$

### Proposition 2

The cdf and hrf of the $\alpha$IW distribution are

$$\begin{aligned} F_{T}(t; \alpha , \beta , \sigma )=&F_{X}(t; \beta , \sigma )+\frac{t}{\alpha }f_{T}(t; \alpha , \beta , \sigma )\\ =&\exp \left( -\sigma t^{-\beta }\right) + \dfrac{\alpha }{\beta \sigma ^{\alpha /\beta }} \Gamma \left( \dfrac{\alpha }{\beta }\right) t^{\alpha } G\left( t^{-\beta }; \dfrac{\alpha }{\beta }+1, \sigma \right) \end{aligned}$$

and

$$\begin{aligned} h_{T}(t; \alpha , \beta , \sigma )=\dfrac{\dfrac{\alpha ^2}{\beta \sigma ^{\alpha /\beta }} \Gamma \left( \dfrac{\alpha }{\beta }\right) t^{\alpha -1} G\left( t^{-\beta }; \dfrac{\alpha }{\beta }+1, \sigma \right) }{1-\left[ \exp \left( -\sigma t^{-\beta }\right) + \dfrac{\alpha }{\beta \sigma ^{\alpha /\beta }} \Gamma \left( \dfrac{\alpha }{\beta }\right) t^{\alpha } G\left( t^{-\beta }; \dfrac{\alpha }{\beta }+1, \sigma \right) \right] }, \end{aligned}$$

respectively.

### Proof

The results follow from the definitions of the $\alpha$-monotone distribution and hrf; see Jones^18^. $\square$

**Figure 1:**
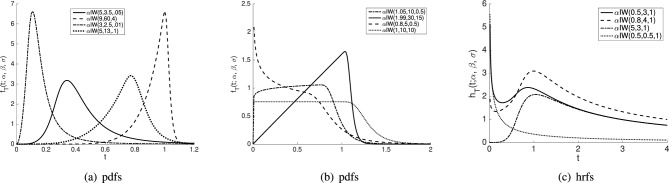
Shapes of the pdf and hrf of the $\alpha$IW distribution for different parameters settings.

The plots for the pdf and hrf of the $\alpha$IW distribution for certain values of the parameters are shown in Fig. 1a,b and c, respectively.

It should be noted that pdf of the $\alpha$IW distirbution can be skewed to the right or left, and also has various shapes for the different parameter settings; see Fig. 1a and b, respectively. Besides, shapes of the hrf of the $\alpha$IW distribution, given in Fig. 1c, can be monotone decrease, monotone decrease-increase (bathup-shape), or monotone increase-decrease. These properties make $\alpha$IW distribution desirable in modeling reliability data.

## Characterization

In this section, the proposed model is characterized by its hrf and characterizing conditions are provided as well.

The pdf of the $\alpha$IW distribution can be expressed as

4 $$\begin{aligned} f(t)&=\alpha \sigma ^{-\frac{\alpha }{\beta }} t^{\alpha -1}\displaystyle \int _{0}^{\sigma t^{-\beta }} u^{\frac{\alpha +\beta }{\beta }-1} \exp \left( -u\right) du\\&=\alpha \sigma ^{\frac{\alpha }{\beta }} t^{\alpha -1}\left[ \Gamma \left( \dfrac{\alpha +\beta }{\beta }\right) - \Gamma \left( \dfrac{\alpha +\beta }{\beta }, \sigma t^{-\beta }\right) \right] , \end{aligned}$$

where $\Gamma (a, z)=\displaystyle \int _{z}^{\infty }u^{a-1} \exp (-u) du$ is an upper incomplete gamma function. Therefore, cdf, survival function, and hrf of the $\alpha$IW distribution can also be expressed as

$$\begin{aligned} F(t)= & {} \exp \left( -\sigma t^{-\beta }\right) + \sigma ^{-\frac{\alpha }{\beta }} t^{\alpha } \left[ \Gamma \left( \frac{\alpha +\beta }{\beta }\right) -\Gamma \left( \frac{\alpha +\beta }{\beta }, \sigma t^{-\beta } \right) \right] , \\ S(t)= & {} {\overline{F}}(t)=1- \exp \left( -\sigma t^{-\beta }\right) - \sigma ^{-\frac{\alpha }{\beta }} t^{\alpha } \left[ \Gamma \left( \frac{\alpha +\beta }{\beta }\right) -\Gamma \left( \frac{\alpha +\beta }{\beta }, \sigma t^{-\beta } \right) \right] , \end{aligned}$$

and

5 $$\begin{aligned} h(t)=\dfrac{f(t)}{{\overline{F}}(t)}=\dfrac{\dfrac{\alpha }{\sigma ^{\alpha /\beta }}t^{\alpha -1}\left[ \Gamma \left( \dfrac{\alpha +\beta }{\beta }\right) - \Gamma \left( \dfrac{\alpha +\beta }{\beta }, \sigma t^{-\beta }\right) \right] }{1- \exp \left( -\sigma t^{-\beta }\right) - t^{\alpha } \sigma ^{-\frac{\alpha }{\beta }}\left[ \Gamma \left( \frac{\alpha +\beta }{\beta }\right) -\Gamma \left( \frac{\alpha +\beta }{\beta }, \sigma t^{-\beta } \right) \right] }, \end{aligned}$$

respectively. Note that the conditions $F(0)=0$ and $F(\infty )=1$ are satisfied, which implies the function *F*(*t*) is a cdf.

### Proposition 3

The random variable $T:\Omega \longrightarrow \left( 0,+\infty \right)$ has continuous pdf *f*(*t*) if and only if the hrf *h*(*t*) satisfies the following equation:

6 $$\begin{aligned} \frac{f'(t)}{f(t)}=\frac{h'(t)}{h(t)}-h(t). \end{aligned}$$

### Proof

According to definition of the hrf, given by in (5), it follows:

$$\begin{aligned} \frac{h'(t)}{h(t)}=\frac{f'(t)\overline{{F}}(t)+f^2(t)}{\overline{{F}}^2(t)}\cdot \frac{\overline{{F}}(t)}{f(t)}=\frac{f'(t)}{f(t)}+h(t). \end{aligned}$$

Thus, the statement of proposition immediately follows. $\square$

### Proposition 4

The random variable $T:\Omega \longrightarrow \left( 0,+\infty \right)$ has $\alpha$IW$(\alpha , \beta , \sigma )$ if and only if the hrf *h*(*t*), defined by (5), satisfies the following equation:

7 $$\begin{aligned} \dfrac{h'(t)}{\left( h(t)\right) ^{2}}=&\dfrac{\left( 1-\dfrac{ \beta \sigma ^{\frac{\alpha }{\beta }+1} t^{-\alpha -\beta } \exp \left( -\sigma t^{-\beta }\right) }{(\alpha -1) \left[ \Gamma \left( \dfrac{\alpha +\beta }{\beta }\right) -\Gamma \left( \dfrac{\alpha +\beta }{\beta }, \sigma t^{-\beta } \right) \right] }\right) }{ \alpha \sigma ^{-\frac{\alpha }{\beta }} t^{\alpha } \left[ \Gamma \left( \dfrac{\alpha +\beta }{\beta }\right) -\Gamma \left( \dfrac{\alpha +\beta }{\beta },t^{-\beta } \sigma \right) \right] }\\&\times \left\{ (\alpha -1) \left[ 1-\exp \left( -\sigma t^{-\beta }\right) - \sigma ^{-\frac{\alpha }{\beta }} t^{\alpha } \left[ \Gamma \left( \dfrac{\alpha +\beta }{\beta }\right) -\Gamma \left( \dfrac{\alpha +\beta }{\beta }, \sigma t^{-\beta } \right) \right] \right] \right\} +1 \end{aligned}$$

### Proof

*Necessity*: Assume that $T\sim \alpha$IW($\alpha , \beta , \sigma$), with the pdf *f*(*t*), defined by (4). Then, natural logarithm of this pdf, can be expressed as:

$$\begin{aligned} \ln \left( f(t)\right) =\ln \alpha - \frac{\alpha }{\beta } \ln \sigma + (\alpha -1) \ln t+ \ln \left[ \Gamma \left( \frac{\alpha +\beta }{\beta }\right) -\Gamma \left( \frac{\alpha +\beta }{\beta }, \sigma t^{-\beta }\right) \right] . \end{aligned}$$

Differentiating both sides of this equality with respect to *t*, we get:

8 $$\begin{aligned} \frac{f'(t)}{f(t)}&=\frac{\alpha -1}{t}\left( 1-\frac{\beta \sigma ^{\frac{\alpha +\beta }{\beta }} t ^{-(\alpha +\beta )} \exp \left( -\sigma t^{-\beta }\right) }{(\alpha -1)\left[ \Gamma \left( \frac{\alpha +\beta }{\beta }\right) -\Gamma \left( \frac{\alpha +\beta }{\beta }, \sigma t^{-\beta }\right) \right] }\right) . \end{aligned}$$

Thus, according to (5), (6), and (8), it follows:

$$\begin{aligned} \frac{h'(t)}{h(t)}&= \frac{\alpha -1}{t}\left( 1-\frac{\beta \sigma ^{\frac{\alpha +\beta }{\beta }} t ^{-(\alpha +\beta )} \exp \left( -\sigma t^{-\beta }\right) }{(\alpha -1)\left[ \Gamma \left( \frac{\alpha +\beta }{\beta }\right) -\Gamma \left( \frac{\alpha +\beta }{\beta }, \sigma t^{-\beta }\right) \right] }\right) \\&\quad +\dfrac{\alpha \sigma ^{-\frac{\alpha }{\beta }}t^{\alpha -1}\left[ \Gamma \left( \dfrac{\alpha +\beta }{\beta }\right) - \Gamma \left( \dfrac{\alpha +\beta }{\beta }, \sigma t^{-\beta }\right) \right] }{1- \exp \left( -\sigma t^{-\beta }\right) - t^{\alpha } \sigma ^{-\frac{\alpha }{\beta }}\left[ \Gamma \left( \frac{\alpha +\beta }{\beta }\right) -\Gamma \left( \frac{\alpha +\beta }{\beta }, \sigma t^{-\beta } \right) \right] }, \end{aligned}$$

which after certain simplification yields (7).

*Sufficiency:* Suppose that (7) holds. After integration, it can be rewritten as follows:

$$\begin{aligned} \int \frac{h'(t)}{\left( h(t)\right) ^{2}} dt=&\int \frac{1}{ \alpha \sigma ^{-\frac{\alpha }{\beta }} t^{\alpha } \left[ \Gamma \left( \frac{\alpha +\beta }{\beta }\right) -\Gamma \left( \frac{\alpha +\beta }{\beta },t^{-\beta } \sigma \right) \right] }\\&\times \Biggl \{ \left[ 1 - \frac{\beta \sigma ^{\frac{\alpha +\beta }{\beta }} t ^{-(\alpha +\beta )} \exp \left( -\sigma t^{-\beta }\right) }{(\alpha -1)\left[ \Gamma \left( \frac{\alpha +\beta }{\beta }\right) -\Gamma \left( \frac{\alpha +\beta }{\beta }, \sigma t^{-\beta }\right) \right] }\right] \\&\times \left[ 1- \exp \left( -\sigma t^{-\beta }\right) - t^{\alpha } \sigma ^{-\frac{\alpha }{\beta }}\left[ \Gamma \left( \frac{\alpha +\beta }{\beta }\right) -\Gamma \left( \frac{\alpha +\beta }{\beta }, \sigma t^{-\beta } \right) \right] \right] (\alpha -1)\\&+ \alpha \sigma ^{-\frac{\alpha }{\beta }}t^{\alpha }\left[ \Gamma \left( \frac{\alpha +\beta }{\beta }\right) - \Gamma \left( \frac{\alpha +\beta }{\beta }, \sigma t^{-\beta }\right) \right] \Biggr \} dt, \end{aligned}$$

from the above differential equation, we have

9 $$\begin{aligned} h(u) =\frac{\alpha \sigma ^{-\frac{\alpha }{\beta }} u^{\alpha -1} \left[ \Gamma \left( \frac{\alpha +\beta }{\beta }\right) - \Gamma \left( \frac{\alpha +\beta }{\beta }, \sigma u^{-\beta }\right) \right] }{1-\exp \left( \sigma u^{-\beta }\right) - \sigma ^{-\frac{\alpha }{\beta }} u^{\alpha } \left[ \Gamma \left( \frac{\alpha +\beta }{\beta }\right) - \Gamma \left( \frac{\alpha +\beta }{\beta }, \sigma u^{-\beta }\right) \right] }. \end{aligned}$$

Integrating (9) from 0 to t, we obtain:

$$\begin{aligned} -\ln (1-F(t))=-\ln \left[ 1- \exp \left( -\sigma t^{-\beta }\right) -\sigma ^{-\frac{\alpha }{\beta }} t^{\alpha } \left( \Gamma \left( \frac{\alpha +\beta }{\beta }\right) - \Gamma \left( \frac{\alpha +\beta }{\beta }, \sigma t^{-\beta }\right) \right) \right] , \end{aligned}$$

which after simplification yields

$$\begin{aligned} F(t)= \exp \left( -\sigma t^{-\beta }\right) + \sigma ^{-\frac{\alpha }{\beta }} t^{\alpha } \left[ \Gamma \left( \frac{\alpha +\beta }{\beta }\right) -\Gamma \left( \frac{\alpha +\beta }{\beta }, \sigma t^{-\beta } \right) \right] \end{aligned}$$

whereby from the conditions $F(0)=0$ and $F(\infty )=1$. Thus, the function *F*(*t*) is indeed the cdf from $\alpha \text {IW}(\alpha ,\beta ,\sigma )$, which completes the proof. $\square$

### Proposition 5

The *r*-th moment of the $\alpha$IW distribution is formulated as follows

$$\begin{aligned} {\textbf{E}}\left[ T^{r}\right]= & {} {\textbf{E}}\left[ X^{r}Y^{\frac{r}{\alpha }}\right] ={\textbf{E}}\left[ X^{r}\right] {\textbf{E}}\left[ Y^{\frac{r}{\alpha }}\right] \\= & {} \sigma ^{r/\beta }\frac{\alpha }{\alpha +r}\Gamma \left( 1-\frac{r}{\beta }\right) ;\quad \frac{r}{\beta }<1. \end{aligned}$$

### Proof

The random variable $T\sim \alpha \text {IW}(\alpha , \beta , \sigma )$ can be expressed by using the stochastic representation $T=X \times Y^{\frac{1}{\alpha }}$. Then,

$$\begin{aligned} {\textbf{E}}\left[ T^{r}\right] ={\textbf{E}}\left[ X^{r} Y^{\frac{r}{\alpha }}\right] ={\textbf{E}}\left[ X^r \right] {\textbf{E}}\left[ Y^{\frac{r}{\alpha }}\right] , \end{aligned}$$

which completes the proof. $\square$

### Proposition 6

The random variable *T,* having pdf given in (3), has an $\alpha$-monotone density since its pdf satisfies the condition

$$\begin{aligned} \frac{d}{dt}(\log f_{T}(t)\le \frac{\alpha -1}{t}, \quad \text {for all }t>0. \end{aligned}$$

### Proof

From the Proposition 1,

$$\begin{aligned} f_{T}(t;\alpha ,\beta ,\sigma )=\displaystyle \int _{0}^{1} \beta \sigma t^{-(\beta +1)} w^{\frac{\beta }{\alpha }}\exp (-\sigma t^{-\beta } w^{\frac{\beta }{\alpha }}) dw. \end{aligned}$$

By using the variable transformation $tw^{-1/\alpha }=u$, $f_{T}(t)$ is expressed as

$$\begin{aligned} f_{T}(t;\alpha ,\beta ,\sigma )&=\int _{t}^{\infty }\alpha t^{\alpha -1} \beta \sigma u^{-(\alpha +\beta +1)}\exp \left( -\sigma u^{-\beta }\right) du\\&=\alpha t^{\alpha -1}\int _{t}^{\infty }\frac{1}{u^\alpha }f_{X}(u;\beta ,\sigma )du.\\ \end{aligned}$$

It is seen that $f_{T}(t)$ can be expressed as

$$\begin{aligned} f_{T}(t)=\alpha t^{\alpha -1}\int _{t}^{\infty }\frac{1}{x^\alpha }f_{X}(x)dx. \end{aligned}$$

Then,

$$\begin{aligned} f^{\prime }_{T}(t)&=\alpha (\alpha -1)t^{\alpha -2}\int _{t}^{\infty }\frac{1}{x^\alpha }f_{X}(x)dx - \left( \alpha t^{\alpha -1}\right) \frac{1}{t^{\alpha }}f_{X}(t)\\&=(\alpha -1)t^{-1}f_{T}(t)-\alpha t^{-1}f_{X}(t)\\ \alpha f_{X}(t)&=\left( \alpha -1\right) f_{T}(t) - t f^{\prime }_{T}(t). \end{aligned}$$

From there, the proof is completed by following lines given below.

$$\begin{aligned} (\alpha -1)f_{T}(t)- t f^{\prime }_{T}(t)&\ge 0 \quad \text {since} \quad f_{X}(t)\ge 0,\\ \dfrac{\alpha -1}{t}&\ge \dfrac{ f^{\prime }_{T}(t)}{f_{T}(t)},\\ \dfrac{\alpha -1}{t}&\ge \frac{d}{dt} \log ( f_{T}(t) ),\\ \frac{d}{dt} \log ( f_{T}(t) )&\le \dfrac{\alpha -1}{t}. \end{aligned}$$

$$\square$$

The $\alpha$IW distribution is obtained as a scale-mixture between the IW and U(0,1) distributions as shown in Proposition 7.

### Proposition 7

Let $T|Y=y\sim \text {IW}(\beta , \sigma y^{\frac{\beta }{\alpha }})$ and $Y\sim \text {U}(0, 1)$, then $T\sim \alpha \text {IW}(\alpha ,\beta ,\sigma )$. Therefore, the $\alpha \text {IW}(\alpha ,\beta , \sigma )$ distribution is a scale-mixture between the $\text {IW}(\beta , \sigma y^{\frac{\beta }{\alpha }})$ and *U*(0, 1) distributions.

### Proof

 

$$\begin{aligned} f_{T}(t;\alpha , \beta , \sigma )&=\int _{0}^{1}f_{Y|U}(t; \beta , \sigma y^{\frac{\beta }{\alpha }})f_{Y}(y)dy\\&=\int _{0}^{1}\beta \sigma y^{\beta /\alpha } t^{-(\beta +1)} \exp \left( -\sigma y^{\beta /\alpha }t^{-\beta }\right) dy \end{aligned}$$

The proof is completed after following the transformation $y^{\beta /\alpha }t^{-\beta }=u$. $\square$

It can be seen from the propositions given above that the $\alpha$-monotone concept is easy to be applied and adds essential propoerties to the baseline distribution, i.e., IW distribution. For example, the cdf of the $\alpha$IW distribution is formed in terms of the cdf of the IW distribution and the pdf of the $\alpha$IW distribution. In addition, the moments of the $\alpha$IW distribution can be easily obtained with the help of the moments of the IW and uniform distributions. Furthermore, the pdf of the $\alpha$IW distribution can be written as a scale-mixture between the IW and uniform distributions and this property may make it attractive in the application.

### Data generation

The steps given below should be followed for obtaining the random variates from the $\alpha \text {IW}(\alpha , \beta , \sigma )$ distribution:

Step 1 Generate random variate *x* from the IW($\alpha , \beta$) distribution via equation
  $$\begin{aligned} x=\left[ -\sigma ^{-1}\ln (p)\right] ^{-1\big /\beta }; \quad 0<p<1,\quad i.e. \quad p\sim \text {U}(0,1). \end{aligned}$$
Step 2 Generate random variate *y* from the U(0,1) distribution.
Step 3 Obtain the random variate from the $\alpha \text {IW}(\alpha , \beta , \sigma )$ distribution via the equation
  $$\begin{aligned} t=x \times y^{1/\alpha }. \end{aligned}$$

Note that steps given above come from the definition for the random variable that follows a $\alpha$IW distribution; see Proposition 1.

## Related distributions

The $\alpha$IW distribution includes some distributions as sub-models, converges to the some other well-known distributions as a limiting case and be slash Weibull distribution under variable transformation. In this section, referred distributions are given briefly.

### Sub-models

Let $T \sim \alpha \text {IW}(\alpha , \beta , \sigma )$, then we have the next submodels.

i. If $\beta =1$, then *T* has an $\alpha$-monotone inverse exponential density given below
  $$\begin{aligned} g(t; \alpha , \sigma )=\dfrac{\alpha ^2}{\sigma ^{\alpha }} \Gamma \left( \alpha \right) t^{\alpha -1} G\left( t^{-1}; \alpha +1, \sigma \right) ;\quad t>0 \quad \alpha , \sigma >0. \end{aligned}$$
ii. If $\beta =2$, then *T* has an $\alpha$-monotone inverse Rayleigh density as follows:
  $$\begin{aligned} g(t; \alpha , \sigma )=\dfrac{\alpha ^2}{2 \sigma ^{\alpha /2}} \Gamma \left( \dfrac{\alpha }{2}\right) t^{\alpha -1} G\left( t^{-2}; \dfrac{\alpha }{2}+1, \sigma \right) ;\quad t>0 \quad \alpha , \sigma >0. \end{aligned}$$

### Limiting distributions

Let $$T \sim \alpha \text {IW}(\alpha , \beta , \sigma )$$.

i. The stochastic representation of the random variable *T* having $\alpha$-monotone distribution is $T=X \times Y^{\frac{1}{\alpha }}$. Therefore, it is trivial that if $\alpha$ goes to infinity, the random variable *T* converenges to the *X*. As a result of this, if $\alpha \rightarrow \infty$, then $\alpha \text {IW}(\alpha , \beta , \sigma )$ converenges to the IW($\beta , \sigma$).
ii. If $\alpha \rightarrow \infty$ and $\beta =1$, then $\alpha \text {IW}(\alpha , \beta , \sigma )$ converenges to the inverse exponential distribution
  $$\begin{aligned} g(t; \sigma )=\sigma t^{-2}\exp \big ({-\sigma t^{-1}}\big ); \quad t>0, \quad \sigma >0. \end{aligned}$$
iii. If $\alpha \rightarrow \infty$ and $\beta =2$, then $\alpha \text {IW}(\alpha , \beta , \sigma )$ converenges to the inverse Rayleigh distribution
  $$\begin{aligned} g(t; \alpha , \sigma )=2 \sigma t^{-3}\exp \big ({-\sigma t^{-2}}\big ); \quad t>0, \quad \sigma >0. \end{aligned}$$

### Under variable transformation

Let $T \sim \alpha \text {IW}(\alpha , \beta , \sigma )$. Then, the random variable *Z* defined by $Z=T^{-1}$ has the pdf

$$\begin{aligned} f_{Z}(z; \alpha , \beta , \sigma ) =\dfrac{\alpha ^2}{\beta \sigma ^{\alpha /\beta }} \Gamma \left( \dfrac{\alpha }{\beta }\right) z^{-(\alpha +1)} G\left( z^{\beta }; \dfrac{\alpha }{\beta }+1, \sigma \right) ; \quad z>0, \quad \alpha , \beta , \sigma >0 \end{aligned}$$

and follows the slash Weibull distribution with a certain reparametrization. Note that slash exponential and slash Rayleigh distributions are sub-models of the slash Weibull distribution.

## Estimation

Let $\underset{\sim }{t}=(t_1, t_2, \cdots , t_n)$ be the observed values of a random sample from $\alpha \text {IW}(\alpha , \beta , \sigma )$ distribution. Then, estimation methods can be used to obtain the estimators of paramaters $\alpha$, $\beta$, and $\sigma$, say ${\hat{\alpha }}, {\hat{\beta }}$, and ${\hat{\sigma }}$, for the $\alpha$IW distribution. In this study, well-known estimation methods maximum likelihood (ML), maximum product of spacing (MPS), and least squares (LS) are considered to obtain estimators of the parameters of the $\alpha$IW distribution. Also, efficiencies of the ML, MPS, and LS estimation methods are compared by conducting a Monte-Carlo simulation study. Note that optimization tools “fminsearch” and “fminunc”, which are available in software MATLAB2015b can be used to find the ML, MPS, and LS estimates of the parameters $\alpha , \beta$, and $\sigma$.

### ML estimation

The idea of the ML method is coming from maximization of the log-likelihood ($\ln L$) function

10 $$\begin{aligned} \ln L\left( \alpha , \beta , \sigma ; \underset{\sim }{t}\right) =2 n\ln \alpha - n \ln \beta - n\left( \frac{\alpha }{\beta }\right) \ln \sigma + n \ln \Gamma \left( \dfrac{\alpha }{\beta }\right) +(\alpha -1)\sum _{i=1}^{n}\ln t_{i} + \sum _{i=1}^{n}\ln \left[ G\left( t_{i}^{-\beta };\frac{\alpha }{\beta }+1,\sigma \right) \right] . \end{aligned}$$

After taking partial derivative of the $\ln L$ function given in (10) with respect to the parameters of interest and setting them equal to 0, the following likelihood equations

11 $$\begin{aligned} \dfrac{\partial \ln L}{\partial \alpha }= & {} \frac{2 n}{\alpha }-\frac{n}{\beta }\ln \sigma + \frac{n}{\beta }\Psi \left( \frac{\alpha }{\beta }\right) +\sum _{i=1}^{n} \ln t_{i}+\sum _{i=1}^{n}\dfrac{\frac{d}{d\alpha }G\left( t_{i}^{-\beta };\frac{\alpha }{\beta }+1,\sigma \right) }{G\left( t_{i}^{-\beta };\frac{\alpha }{\beta }+1,\sigma \right) }=0 \quad , \end{aligned}$$

12 $$\begin{aligned} \dfrac{\partial \ln L}{\partial \beta }= & {} n\frac{\alpha }{\beta ^2}\ln \sigma - \frac{n}{\beta }-n\frac{\alpha }{\beta ^2}\Psi \left( \frac{\alpha }{\beta }\right) +\sum _{i=1}^{n}\dfrac{\frac{d}{d\beta }G\left( t_{i}^{-\beta };\frac{\alpha }{\beta }+1,\sigma \right) }{G\left( t_{i}^{-\beta };\frac{\alpha }{\beta }+1,\sigma \right) }=0 \quad , \end{aligned}$$

and

13 $$\begin{aligned} \dfrac{\partial \ln L}{\partial \sigma }=-n\frac{\alpha }{\beta \sigma } +\sum _{i=1}^{n}\dfrac{\frac{d}{d\sigma }G\left( t_{i}^{-\beta };\frac{\alpha }{\beta }+1,\sigma \right) }{G\left( t_{i}^{-\beta };\frac{\alpha }{\beta }+1,\sigma \right) }=0 \end{aligned}$$

are obtianed. Here, $\Psi {(\cdot )}$ represents the digamma function. Since likelihood Eqs. (11–13) include nonlinear functions of the parameters $\alpha$, $\beta$ and $\sigma$, they cannot be solved explicitly. Therefore, the ML estimates of the parameters $\alpha$, $\beta$, and $\sigma$, say ${\hat{\alpha }}_{ML}$, ${\hat{\beta }}_{ML}$, and ${\hat{\sigma }}_{ML}$, are obtained by solving likelihood equations (11–13) simulatanously. Note that the ML estimators has approximately a $N_{3}(\Theta , \varvec{I^{-1}(\Theta )})$ distribution where $\varvec{I(\Theta )}$ is the expected information matrix. However, the matrix $\varvec{J(\Theta )}$, which is equal to $-\varvec{H}(\Theta )$ and $\varvec{H}$ denotes the Hessian matrix, evaluated at ${\hat{\Theta }}$ can be used if the matrix $\varvec{I(\Theta )}$ for $\Theta$ can not be obtained explicitly. Therefore, asymptotic confidence intervals for the parameters $\alpha , \beta$, and $\sigma$ are defined by using the matrix $\varvec{J}(\Theta )$, where $\Theta =(\alpha , \beta , \sigma )^{\top }$. The entries of $\varvec{H}$ are given in the Supplementary Material of the paper as an appendix. Also, ML estimation of the parameters $\alpha$, $\beta$, and $\sigma$ is considered under progressive Type-II censored sample and provided in the Supplementary Material of the paper as an appendix.

### MPS estimation

The MPS estimates of the parameters $\alpha$, $\beta$, and $\sigma$, say ${\hat{\alpha }}_{MPS}$, ${\hat{\beta }}_{MPS}$, and ${\hat{\sigma }}_{MPS}$, of the $\alpha$IW distribution are the points in which the objective function

$$\begin{aligned}MPS\left( \alpha , \beta , \sigma ; \underset{\sim }{t}\right)&= \left( \dfrac{1}{n+1}\right) \sum _{i=0}^{n} \ln \bigg [ \exp \left( -\sigma t_{(i+1)}^{-\beta }\right) + \dfrac{\alpha }{\beta \sigma ^{\alpha /\beta }} \Gamma \left( \dfrac{\alpha }{\beta }\right) t_{(i+1)}^{\alpha } G\left( t_{i+1}^{-\beta }; \dfrac{\alpha }{\beta }+1, \sigma \right) \\&\quad -\exp \left( -\sigma t_{(i)}^{-\beta }\right) + \dfrac{\alpha }{\beta \sigma ^{\alpha /\beta }} \Gamma \left( \dfrac{\alpha }{\beta }\right) t_{(i)}^{\alpha } G\left( t_{(i)}^{-\beta }; \dfrac{\alpha }{\beta }+1, \sigma \right) \bigg ] \end{aligned}$$

attains its maximum. Here, $t_{(\cdot )}$ denotes ordered observation, i.e., $t_{(1)} \le t_{(2)} \le \cdots \le t_{(n-1)} \le t_{(n)}$. Note that $t_{0}$ and $t_{(n+1)}$ are the values in which $F_{T}(t_{0}; \alpha , \beta , \sigma )\equiv 0$ and $F_{T}(t_{n+1}; \alpha , \beta , \sigma )\equiv 1$; see Bagci et al.^22^ and references given there in.

### LS estimation

In the LS method, for the case of the $\alpha$IW distribution, it is aimed to minimize the objective function

$$\begin{aligned} LS\left( \alpha , \beta , \sigma ; \underset{\sim }{t}\right) =&\dfrac{1}{n}\sum _{i=1}^{n}\left[ F_{T}(t_{(i); \alpha , \beta , \sigma })- \dfrac{i}{n+1}\right] ^2\\&\dfrac{1}{n}\sum _{i=1}^{n}\left[ \left( \exp \left( -\sigma t_{(i)}^{-\beta }\right) + \dfrac{\alpha }{\beta \sigma ^{\alpha /\beta }} \Gamma \left( \dfrac{\alpha }{\beta }\right) t_{(i)}^{\alpha } G\left( t_{(i)}^{-\beta }; \dfrac{\alpha }{\beta }+1, \sigma \right) \right) -\left( \frac{i}{n+1}\right) \right] ^2 \end{aligned}$$

with respect to the parameters of interest ($\alpha$, $\beta$, and $\sigma$) and the results are called the LS estimates of the parameters $\alpha$, $\beta$, and $\sigma$, i.e., ${\hat{\alpha }}_{LS}$, ${\hat{\beta }}_{LS}$, and ${\hat{\sigma }}_{MPS}$; see Acitas and Arslan^23^ and references therein for further information.

### Monte-Carlo simulation

In this subsection, a Monte-Carlo simulation study is conducted to compare the efficiencies of the ML, MPS, and LS estimation methods. Random samples are generated from the $\alpha$IW distribution, as presented in the data generation subsection, for the sample sizes 100, 200, and 300 and different parameter settings; see Table 1.

**Table 1:** Parameter settings considered in the simulation.

Parameter	Scenario							
	1	2	3	4	5	6	7	8
$\alpha$	0.8	0.7	1.5	0.9	2.0	1.7	1.0	2.0
$\beta$	0.5	0.9	0.8	1.7	1.5	2.5	1.0	2.0
$\sigma$	1.0	1.0	1.0	1.0	1.0	1.0	1.0	1.0

Note that the scale parameter $\sigma$ is taken to be 1 througout all simulation scenario without loss of generality. All the simulations are conducted for $\lfloor 100,000/n \rfloor$ Monte-Carlo runs, where $\lfloor \cdot \rfloor$ denotes the integer value function via MATLAB2015b. The ML, MPS, and LS estimates of the parameters are obtained by using the optimization tools “fminunc”, which is available in software MATLAB2015b. Efficiencies of the ML, MPS, and LS methods are compared by using bias, variance, and mean squared error (MSE) criteria. The simulation results are given in Table 2 and summarized as follows.

- Scenario 1: The ML method gives the smallest bias values for $\alpha$ in all sample sizes. However, in terms of the MSE criterion, the MPS method results the smallest values for $\alpha$ in all sample sizes. Concerning the $\beta$, and $\sigma$, the ML, MPS, and LS methods have negligible biases and small variances for all sample sizes *n*.
- Scenario 2: The ML, MPS and LS methods have the negligible bias values and small variances for the $\alpha$, and $\beta$ for all sample sizes; however the LS method gives the largest MSE values for $\sigma$. When $n=300$, the ML, MPS, and LS show more or less the same performances.
- Scenario 3: The MPS method has the largest bias values and the LS gives the largest MSE values for $\alpha$ in all sample sizes. However, the ML and MPS methods give more or less the same bias and the MSE values for $\beta$ and $\sigma$ for all sample sizes.
- Scenario 4: The ML, MPS, and LS methods result negligible bias and small MSE values for $\alpha$, $\beta$, and $\sigma$ when sample size $n=200$ and $n=300$. However, the ML, MPS, and LS do not show the good performances for $\sigma$ in the sample size $n=100$.
- Scenario 5: The LS method gives the biggest MSE values for $\alpha$ $\beta$, and $\sigma$ in all sample sizes. The ML, MPS and LS methods have the small bias values and small variances for the $\alpha$, $\beta$ and $\sigma$ for the sample size $n=200$ and $n=300$.
- Scenario 6: The MPS method produces smallest bias and MSE values for $\alpha$, $\beta$, and $\sigma$ when the sample size $n=100$. The ML, MPS, and LS methods show more or less the same performances for $\alpha$, $\beta$, and $\sigma$ when the sample size $n=300$.
- Scenario 7: The ML, MPS, and LS methods have negligible bias values for $\alpha$ and $\beta$; however the LS gives the largest MSE values for $\alpha$ and $\sigma$, except the sample size $n=300$. The MPS and ML have similar efficiencies for $\alpha$, $\beta$, and $\sigma$ for all sample sizes *n*.
- Scenario 8: The LS method shows the worst performance for $\alpha$ when the sample $n=100$. Concerning the $\beta$ and $\sigma$, the ML, MPS, and LS methods produce more or less the same results for sample sizes $n=200$ and $n=300$.

To sum up, the ML and MPS methods are very closed easch other and stand one step ahead of the LS method. It should also be noted that the simulated MSE values for each parameter of the ML, MPS, and LS estimates are decreasing when the sample size increases, as expected.

**Table 2:** The simulated bias, variance, and MSE values of the ML, MPS, and LS estimates.

n		ML			MPS			LS			ML			MPS			LS		
		Bias	Variance	MSE	Bias	Variance	MSE	Bias	Variance	MSE	Bias	Variance	MSE	Bias	Variance	MSE	Bias	Variance	MSE
		$\alpha =0.8, \quad \beta =0.5, \quad \sigma =1.0$									$\alpha =0.7, \quad \beta =0.9, \quad \sigma =1.0$								
100	${\hat{\alpha }}$	− 0.0176	0.0709	0.0711	0.0854	0.0261	0.0333	0.0054	0.1210	0.1209	− 0.0362	0.0269	0.0282	0.0076	0.0193	0.0193	− 0.0450	0.0537	0.0557
	${\hat{\beta }}$	− 0.0334	0.0051	0.0062	− 0.0228	0.0045	0.0050	− 0.0401	0.0075	0.0091	− 0.0449	0.0214	0.0234	− 0.0115	0.0182	0.0183	− 0.0401	0.0279	0.0295
	${\hat{\sigma }}$	− 0.0896	0.0770	0.0849	− 0.1543	0.0732	0.0969	− 0.1860	0.1655	0.1999	− 0.0615	0.1063	0.1100	− 0.0966	0.0927	0.1019	− 0.0839	0.1328	0.1397
200	${\hat{\alpha }}$	− 0.0132	0.0309	0.0311	0.0452	0.0187	0.0207	− 0.0325	0.1013	0.1021	− 0.0092	0.0100	0.0101	0.0138	0.0087	0.0089	− 0.0124	0.0152	0.0154
	${\hat{\beta }}$	− 0.0223	0.0028	0.0033	− 0.0172	0.0026	0.0029	− 0.0256	0.0035	0.0042	− 0.0324	0.0133	0.0143	− 0.0141	0.0121	0.0122	− 0.0270	0.0158	0.0165
	${\hat{\sigma }}$	− 0.0439	0.0295	0.0313	− 0.0837	0.0291	0.0361	− 0.0853	0.0627	0.0698	− 0.0491	0.0520	0.0543	− 0.0707	0.0481	0.0530	− 0.0561	0.0612	0.0642
300	${\hat{\alpha }}$	0.0020	0.0203	0.0202	0.0421	0.0145	0.0162	− 0.0372	0.0882	0.0893	− 0.0093	0.0082	0.0083	0.0070	0.0075	0.0075	− 0.0074	0.0112	0.0112
	${\hat{\beta }}$	− 0.0188	0.0024	0.0028	− 0.0153	0.0023	0.0026	− 0.0182	0.0033	0.0036	− 0.0253	0.0079	0.0086	− 0.0126	0.0075	0.0076	− 0.0226	0.0095	0.0100
	${\hat{\sigma }}$	− 0.0424	0.0222	0.0239	− 0.0708	0.0220	0.0269	− 0.0553	0.0389	0.0418	− 0.0270	0.0255	0.0261	− 0.0432	0.0250	0.0268	− 0.0386	0.0349	0.0363

		$\alpha =1.5, \quad \beta =0.8, \quad \sigma =1.0$									$\alpha =0.9, \quad \beta =1.7, \quad \sigma =1.0$								
100	${\hat{\alpha }}$	0.0655	0.1760	0.1801	0.2599	0.0695	0.1370	0.0791	0.4606	0.4664	− 0.0417	0.0261	0.0278	0.0007	0.0212	0.0212	− 0.0377	0.0316	0.0330
	${\hat{\beta }}$	− 0.0551	0.0130	0.0160	− 0.0402	0.0116	0.0132	− 0.0652	0.0179	0.0221	− 0.0752	0.1259	0.1314	0.0117	0.0904	0.0905	− 0.0510	0.1268	0.1293
	${\hat{\sigma }}$	− 0.1203	0.0902	0.1046	− 0.1923	0.0852	0.1221	− 0.2227	0.2019	0.2513	− 0.0701	0.3988	0.4033	− 0.0757	0.1838	0.1893	− 0.0597	0.1599	0.1633
200	${\hat{\alpha }}$	0.0754	0.0839	0.0894	0.1853	0.0509	0.0851	0.0260	0.3255	0.3255	− 0.0073	0.0097	0.0097	0.0145	0.0088	0.0090	− 0.0026	0.0124	0.0124
	${\hat{\beta }}$	− 0.0440	0.0066	0.0086	− 0.0367	0.0063	0.0077	− 0.0477	0.0088	0.0110	− 0.0420	0.0443	0.0460	0.0046	0.0393	0.0392	− 0.0332	0.0624	0.0634
	${\hat{\sigma }}$	− 0.0803	0.0314	0.0378	− 0.1234	0.0313	0.0465	− 0.1115	0.0549	0.0672	− 0.0368	0.0563	0.0575	− 0.0497	0.0509	0.0533	− 0.0467	0.0589	0.0610
300	${\hat{\alpha }}$	0.0403	0.0723	0.0737	0.1282	0.0481	0.0644	0.0003	0.2943	0.2934	− 0.0195	0.0066	0.0070	− 0.0034	0.0062	0.0062	− 0.0229	0.0083	0.0088
	${\hat{\beta }}$	− 0.0359	0.0066	0.0079	− 0.0317	0.0064	0.0074	− 0.0393	0.0085	0.0100	− 0.0152	0.0249	0.0250	0.0163	0.0230	0.0232	− 0.0032	0.0320	0.0319
	${\hat{\sigma }}$	− 0.0478	0.0221	0.0244	− 0.0802	0.0221	0.0285	− 0.0836	0.0459	0.0527	− 0.0043	0.0266	0.0265	− 0.0152	0.0255	0.0256	− 0.0014	0.0280	0.0279

n		$\alpha =2.0, \quad \beta =1.5, \quad \sigma =1.0$									$\alpha =1.7, \quad \beta =2.5, \quad \sigma =1.0$								
100	${\hat{\alpha }}$	0.0098	0.2428	0.2426	0.1949	0.1347	0.1725	− 0.0210	0.6657	0.6655	− 0.0578	0.1064	0.1097	0.0328	0.0842	0.0851	− 0.0559	0.1826	0.1855
	${\hat{\beta }}$	− 0.0928	0.0476	0.0561	− 0.0551	0.0424	0.0454	− 0.0932	0.0646	0.0732	− 0.1147	0.1901	0.2030	− 0.0102	0.1564	0.1563	− 0.0888	0.2441	0.2518
	${\hat{\sigma }}$	− 0.1020	0.0775	0.0878	− 0.1578	0.0744	0.0992	− 0.1601	0.1406	0.1661	− 0.0844	0.1372	0.1441	− 0.1113	0.1164	0.1287	− 0.1163	0.2211	0.2344
200	${\hat{\alpha }}$	0.0131	0.1189	0.1189	0.1179	0.0885	0.1022	− 0.0196	0.4118	0.4114	− 0.0097	0.0565	0.0565	0.0391	0.0504	0.0518	− 0.0143	0.0773	0.0774
	${\hat{\beta }}$	− 0.0692	0.0243	0.0290	− 0.0501	0.0228	0.0253	− 0.0707	0.0308	0.0358	− 0.1184	0.0902	0.1040	− 0.0601	0.0816	0.0850	− 0.0984	0.1162	0.1256
	${\hat{\sigma }}$	− 0.0555	0.0419	0.0449	− 0.0892	0.0418	0.0497	− 0.0850	0.0667	0.0738	− 0.0766	0.0628	0.0685	− 0.0948	0.0598	0.0687	− 0.0800	0.0707	0.0770
300	${\hat{\alpha }}$	0.0639	0.0689	0.0728	0.1356	0.0565	0.0748	0.0129	0.2596	0.2590	− 0.0096	0.0366	0.0366	0.0250	0.0335	0.0341	− 0.0041	0.0505	0.0504
	${\hat{\beta }}$	− 0.0588	0.0190	0.0224	− 0.0455	0.0181	0.0201	− 0.0613	0.0285	0.0322	− 0.0722	0.0577	0.0627	− 0.0322	0.0541	0.0549	− 0.0750	0.0800	0.0854
	${\hat{\sigma }}$	− 0.0533	0.0293	0.0321	− 0.0775	0.0293	0.0352	− 0.0701	0.0523	0.0571	− 0.0364	0.0313	0.0325	− 0.0500	0.0305	0.0329	− 0.0516	0.0417	0.0442

n		$\alpha =1.0, \quad \beta =1.0, \quad \sigma =1.0$									$\alpha =2.0, \quad \beta =2.0, \quad \sigma =1.0$								
100	${\hat{\alpha }}$	− 0.0228	0.0524	0.0529	0.0507	0.0347	0.0372	− 0.0323	0.1256	0.1265	− 0.1605	0.3268	0.3523	0.0142	0.1918	0.1918	− 0.1837	0.6187	0.6519
	${\hat{\beta }}$	− 0.0501	0.0240	0.0265	− 0.0191	0.0206	0.0209	− 0.0476	0.0329	0.0351	− 0.0582	0.0870	0.0903	− 0.0008	0.0745	0.0744	− 0.0508	0.1086	0.1111
	${\hat{\sigma }}$	− 0.0788	0.0931	0.0992	− 0.1224	0.0837	0.0986	− 0.1185	0.1388	0.1527	− 0.0328	0.1048	0.1058	− 0.0785	0.0925	0.0985	− 0.0633	0.1407	0.1446
200	${\hat{\alpha }}$	− 0.0434	0.0420	0.0438	0.0013	0.0312	0.0311	− 0.0523	0.0770	0.0796	− 0.0800	0.1244	0.1306	0.0086	0.0935	0.0934	− 0.0642	0.1895	0.1932
	${\hat{\beta }}$	− 0.0269	0.0147	0.0154	− 0.0111	0.0137	0.0138	− 0.0244	0.0176	0.0181	− 0.0428	0.0477	0.0495	− 0.0118	0.0441	0.0441	− 0.0415	0.0583	0.0599
	${\hat{\sigma }}$	− 0.0200	0.0474	0.0477	− 0.0473	0.0467	0.0488	− 0.0361	0.0632	0.0644	− 0.0224	0.0418	0.0422	− 0.0502	0.0412	0.0436	− 0.0458	0.0546	0.0566
300	${\hat{\alpha }}$	− 0.0338	0.0234	0.0245	− 0.0031	0.0203	0.0203	− 0.0433	0.0483	0.0501	− 0.0518	0.0694	0.0719	0.0094	0.0598	0.0597	− 0.0821	0.1448	0.1511
	${\hat{\beta }}$	− 0.0150	0.0107	0.0109	− 0.0040	0.0102	0.0101	− 0.0189	0.0133	0.0136	− 0.0388	0.0321	0.0335	− 0.0176	0.0304	0.0306	− 0.0232	0.0383	0.0387
	${\hat{\sigma }}$	− 0.0061	0.0242	0.0242	− 0.0259	0.0239	0.0245	− 0.0191	0.0365	0.0368	− 0.0135	0.0279	0.0280	− 0.0340	0.0277	0.0288	− 0.0140	0.0348	0.0349

## Applications

In this section, $\alpha$IW distribution is used to model two popular data sets,called Kevlar 49/epoxy and Kevlar 373/epoxy, from the related literature.

There exist various extended/generalized versions of the IW distribution in the literature. To the best of authors' knowledge, Kumaraswamy inverse Weibull (KIW)^24^, alpha power inverse Weibull (APIW)^25^, Marshall-Olkin extended inverse Weibull (MOEIW)^26^, and exponentiated exponential inverse Weibull (EEIW)^27^ distributions, which can be strong alternative to the $\alpha$IW distribution, have not been used in modeling Kevlar 49/epoxy and Kevlar 371/epoxy data sets. Therefore, the modeling performance of the $\alpha$IW distribution is compared with the KIW, APIW, MOEIW, and EEIW distributions. In the comparisons, the well known information criteria the corrected Akaike Information Criterion (AICc), Bayesian Information Criterion (BIC), and the following goodness-of-fit statistics; Kolmogorov-Smirnov (KS), Anderson-Darling (AD), and Cramér-von Misses (CvM) are considered. In the estimation, the ML, MPS, and LS methods are used to obtain estimates of the parameters of the $\alpha$IW distribution. The ML method is considered for the KIW, APIW, MOEIW, EEIW distributions. The optimization tool “fminunc” and “fminsearch” available in software MATLAB2015b is utilised to obtain the corresponding estimates of the parameters of the $\alpha$IW, IW, KIW, APIW, MOEIW, and EEIW distributions.

### Application-I

The Kevlar 49/epoxy data set, given in Table 3, involves stress-rupture life of kevlar 49/epoxy strands subjecting to constant sustained pressure at the 90% stress level until the all had failed; see Andrews and Herzberg^28^.

**Table 3:** Kevlar 49/epoxy data ($n=101$).

0.01	0.01	0.02	0.02	0.02	0.03	0.03	0.04	0.05	0.06	0.07	0.07	0.08	0.09	0.09	0.10	0.10
0.11	0.11	0.12	0.13	0.18	0.19	0.20	0.23	0.24	0.24	0.29	0.34	0.35	0.36	0.38	0.40	0.42
0.43	0.52	0.54	0.56	0.60	0.60	0.63	0.65	0.67	0.68	0.72	0.72	0.72	0.73	0.79	0.79	0.80
1.450	0.80	1.50	0.83	1.51	0.85	1.52	0.90	1.53	0.92	1.54	0.95	1.54	0.99	1.55	1.00	1.58
1.01	1.60	1.02	1.63	1.03	1.64	1.05	1.80	1.10	1.80	1.10	1.81	1.11	2.02	1.15	2.05	1.18
2.14	1.20	2.17	1.29	2.33	1.31	3.03	1.33	3.03	1.34	3.34	1.40	4.20	1.43	4.69	7.89	

Many authors proposed different distributions to model the Kevlar 49/epoxy data. For example, Paranaiba et al.^29^ derived Kumaraswamy Burr XII distribution, Olmos et al.^30^ derived slash generalized half-normal distribution, and Sen et al.^31^ introduced quasi Xgamma-Poisson (QXGP) distribution to model Kevlar 49/epoxy. The QXGP distribution modeled Kevlar 49/epoxy data better than the distributions formerly used and also recently proposed by the others. Therefore, in modeling Kevlar 49/epoxy, the QXGP distribution is also considered besides with the IKW, APIW, MOEIW, and EEIW for the sake of completeness of comparisons. The estimates of the parameters of the $\alpha$IW, IW, QXGP, KIW, APIW, MOEIW, and EEIW distributions and the corresponding fitting results are given in Table 4.

**Table 4:** Fitting results for the Kevlar 49/epoxy data.

Distribution		Parameter estimates				Information criteria			Goodness-of-fit statistics		
$\alpha$IW			${\hat{\alpha }}$	${\hat{\beta }}$	${\hat{\sigma }}$	${\ln L}$	*AICc*	*BIC*	KS	AD	CvM
	ML	–	0.6976	2.9919	5.8426	– 99.2187	204.6849	212.2828	0.0626	0.4058	0.0563
	MPS	–	0.6805	2.7261	4.9866	–	–	–	0.0565	0.4121	0.0615
	LS	–	0.6536	4.2615	19.5937	–	–	–	0.0523	0.4394	0.0436
IW				${\hat{\beta }}$	${\hat{\sigma }}$	${\ln L}$	*AICc*	*BIC*	KS	AD	CvM
	ML	–	–	0.6132	0.4195	− 132.4394	269.0013	274.1091	0.1816	6.0910	1.1054
QXGP			${\hat{\alpha }}$	${\hat{\theta }}$	${\hat{\lambda }}$	${\ln L}$	*AICc*	*BIC*	KS	AD	CvM
	ML	—	0.3065	1.0051	4.4307	– 101.1425	208.5324	216.1304	0.0839	0.9528	0.1168
KIW		${\hat{a}}$	${\hat{b}}$	${\hat{\alpha }}$	${\hat{\beta }}$	${\ln L}$	*AICc*	*BIC*	KS	AD	CvM
	ML	3.3567	8.2904.3458	3.3575	0.0781	– 104.2951	217.0069	227.0507	0.1110	1.6485	0.2982
APIW			${\hat{\alpha }}$	${\hat{\beta }}$	${\hat{\lambda }}$	${\ln L}$	*AICc*	*BIC*	KS	AD	CvM
	ML	–	43.2495	0.7790	0.1158	– 124.3660	254.9794	262.5773	0.1639	4.6086	0.7799
MOEIW			${\hat{\alpha }}$	${\hat{\beta }}$	${\hat{\theta }}$	${\ln L}$	*AICc*	*BIC*	KS	AD	CvM
	ML	–	0.0018	1.2632	307.8233	– 243.6369	493.5211	501.1191	0.1121	2.8377	0.3959
EEIW			${\hat{\alpha }}$	${\hat{\beta }}$	${\hat{c}}$	${\ln L}$	*AICc*	*BIC*	KS	AD	CvM
	ML	–	0.0922	13271.9733	9.4313	– 104.7061	215.6597	223.2576	0.1147	1.7591	0.3188

**Figure 2:**
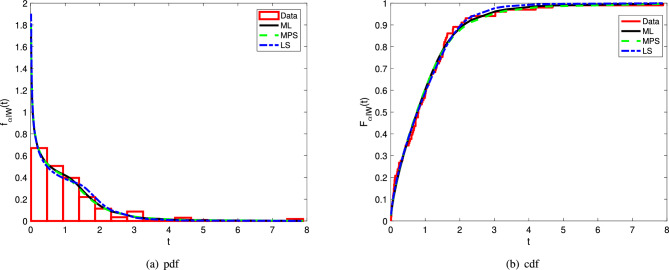
The pdf and cdf plots of the $\alpha$IW with histogram and empirical cdf of the Kevlar 49/epoxy data.

The results given in Table 4 show that the $\alpha$IW distribution performs better modeling perfomance than the QXGP, IW, KIW, APIW, MOEIW, and EEIW distributions when the AICc, BIC, KS, AD, and CvM criteria are taken into account. The fitting performance of the $\alpha$IW distribution is also illustrated graphically by Fig. 2.

It is seen from Fig. 2 that the pdf and cdf of the $\alpha$IW distribution preciously fit the histogram and emprical cdf of the Kevlar 49/epoxy, respectively. This conclusion is also supported by values of the information criteria and goodness-of-fit statistics given in Table 4. Moreover, the $\alpha$IW attains this conclusion when compared to the other competitive distributions and with a considerably bigger difference as well, as per Raftery^32^, which states that the significant difference in the BIC of the models should be more than 2. Also, note that the ML, MPS, and LS estimation methods give more or less the same fitting performances; see the corresponding goodness-of-fit statistics given in Table 4.

### Application-II

The Kevlar 373/epoxy data set involves life of fatigue fracture of kevlar 373/epoxy subjecting to constant pressure at the 90% stress level until all had failed, therefore it includes the exact times of failure. This data set was provided in Glaser^33^; see also Table 5.

**Table 5:** Kevlar 373/epoxy data ($n=76$).

0.0251	0.0886	0.0891	0.2501	0.3113	0.6566	0.6748	0.6751	0.6753	0.7696	0.8851	0.9113
0.9120	0.9836	1.0483	1.2766	1.2985	1.3211	1.3503	1.3551	1.7083	1.7263	1.7460	1.7630
1.7746	1.8878	1.8881	1.9316	1.9558	2.0048	2.2100	2.2460	2.2878	2.3203	2.3470	3.0256
3.2678	3.4045	3.4846	3.7433	5.4435	5.5295	6.5541	9.0960	0.3451	0.4763	0.5650	0.8375
0.8391	0.8425	1.0596	1.0773	1.1733	1.4595	1.4880	1.5728	1.8275	1.8375	1.8503	2.0408
2.0903	2.1093	2.3513	2.4951	2.5260	3.7455	3.9143	4.8073	0.5671	0.8645	1.2570	1.5733
1.8808	2.1330	2.9911	5.4005								

In the literature, there exist various distributions used in modeling Kevler 373/epoxy data. For example, Merovci et al.^34^ proposed generalized transmuted exponential distribution, Alizadeh et al.^35^ proposed an other generalized transmuted exponential distribution, Dey et al.^36^ introduced alpha power transformed Weibull distribution, and Jamal and Chesneau^37^ proposed transformation of Weibull distribution using sine and cosine functions (TSCW) to model Kevlar 373/epoxy. Note that TSCW distribution introduced by Jamal and Chesneau^37^ is preferable over among the others, since it has smaller values of the goodness-of-fit statistics. Therefore, besides with the KIW, APIW, MOEIW, and EEIW distributions, the TSCW distribution is also taken into account to make comparisons complete. The ML estimates of the parameters for the $\alpha$IW, IW, TSCW, KIW, APIW, MOEIW, and EEIW distributions, and the corresponding fitting results are given in Table 6. Also, the MPS and LS estimates of the parameters of the $\alpha$IW distribution along with the corresponding goodness-of-fit statistcs are given in Table 6.

**Table 6:** Fitting results for the Kevlar 373/epoxy data.

Distribution		Parameter estimates				Information criteria			Goodness-of-fit statistics		
$\alpha$IW			${\hat{\alpha }}$	${\hat{\beta }}$	${\hat{\sigma }}$	${\ln L}$	*AICc*	*BIC*	KS	AD	CvM
	ML	–	1.2569	2.8183	13.6493	– 120.0904	246.5141	253.1730	0.0626	0.2835	0.0417
	MPS	–	1.1841	2.6486	12.2142	–	–	–	0.0696	0.3511	0.0572
	LS	–	1.3071	2.8255	13.1439	–	–	–	0.0605	0.2750	0.0385
IW				${\hat{\beta }}$	${\hat{\sigma }}$	${\ln L}$	*AICc*	*BIC*	KS	AD	CvM
	ML	–	–	0.7588	0.8608	– 153.5392	311.2428	315.7399	0.1893	6.4668	1.1195
TSCW			${\hat{\alpha }}$	${\hat{\theta }}$	${\hat{\lambda }}$	${\ln L}$	*AICc*	*BIC*	KS	AD	CvM
	ML	–	0.1492	1.5292	0.1304	– 120.4692	247.2717	253.9306	0.0748	0.4177	0.0694
KIW		${\hat{a}}$	${\hat{b}}$	${\hat{\alpha }}$	${\hat{\beta }}$	${\ln L}$	*AICc*	*BIC*	KS	AD	CvM
	ML	3.0849	52235.8742	3.0849	0.1495	– 123.0827	254.7288	263.4884	0.0893	0.7673	0.1263
APIW			${\hat{\alpha }}$	${\hat{\beta }}$	${\hat{\lambda }}$	${\ln L}$	*AICc*	*BIC*	KS	AD	CvM
	ML	–	813.2749	1.0528	0.1596	– 140.2782	286.8897	293.5486	0.1421	3.5709	0.5474
MOEIW			${\hat{\alpha }}$	${\hat{\beta }}$	${\hat{\theta }}$	${\ln L}$	*AICc*	*BIC*	KS	AD	CvM
	ML	–	0.0002	2.0759	15768.9516	– 124.2789	254.8911	261.5499	0.0839	0.7261	0.0905
EEIW			${\hat{\alpha }}$	${\hat{\beta }}$	${\hat{c}}$	${\ln L}$	*AICc*	*BIC*	KS	AD	CvM
	ML	–	0.1970	565.7956	7.2675	– 123.8862	254.1057	260.7645	0.0849	0.8712	0.1434

**Figure 3:**
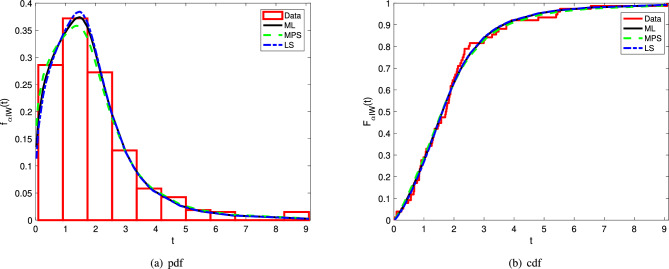
The pdf and cdf plots of the $\alpha$IW with histogram and empirical cdf of the Kevlar 373/epoxy data.

When taking into account the KS, AD, and CvM values, it can be seen from Table 6 that the $\alpha$IW distribution is preferable to the IW, KIW, APIW, MOEIW, and EEIW distributions. The $\alpha$IW and TSCW distributions show more or less the same modeling performace based on the infromation criteria; however, the $\alpha$IW stands one step ahead of the TSCW distribution when goodness-of-fit statistics are taken into account.The fitting performance of the $\alpha$IW distribution is also illustrated in Fig. 3.

Note that the results given in Table 6 is also supported by the Fig. 3, i.e., the density and cdf of the $\alpha$IW distribution preciously fit the histogram and emprical cdf of the Kevlar 373/epoxy, respectively.

## Conclusion

In this study, the $\alpha$IW distribution is derived as a scale-mixture between IW and U(0, 1) distributions. Some statistical properties of the $\alpha$IW distribution are provided. It should be noted that $\alpha$-monotone inverse Rayleigh, $\alpha$-monotone inverse exponential distributions are sub-models of the $\alpha$IW distribution, and also IW, inverse Rayleigh, and inverse exponential distributions are limiting distributions of the $\alpha$IW distribution for the different parameters settings.

The $\alpha$IW distribution is used to model two popular data sets from the reliability area, i.e. Kevlar 49/epoxy and Kevlar 373/epoxy data sets. Literature review show that QXGP distribution proposed by Sen et al.^31^ and TSCW distribution introduced by Jamal and Chesneau^37^ are modeled the Kevlar 49/epoxy and Kevlar 373/epoxy data better than the formerly used distributions, respectively. Therefore, modeling capability of the $\alpha$IW distribution is compared with the modeling capability of the QXGP and TSCW distributions for the corresponding data set. It should be noted IW, KIW, APIW, MOEIW, and EEIW distributions are also included to the comparisons to make the study complete. In the comparisons, AICc, BIC, KS, AD, and CvM criteria are used. Results show that the $\alpha$IW distribution performs better fitting performance than the QXGP, TSCW, KIW, APIW, MOEIW, and EEIW distributions and therewithal the other distributions that are formerly used in modeling these data sets.

The results in Tables 4 and 6 show that the $\alpha$-monotone concept significantly contributes to increase the modeling performance of the baseline distribution, i.e., IW distribution. Thus, obtaining the $\alpha$IW distribution by using the $\alpha$-monotone concept is cost effective. In other words, the new shape parameter added to the distribution by using the $\alpha$-monotone concept significantly increases the modeling capability of the IW distribution. As a result of this study, it is shown that the $\alpha$IW distribution can be an alternative to the well-known and recently-introduced distributions for modeling purposes.

It is known that censored samples may be occured in lifetime analysis and there are many other estimaiton methods, such as method of moments, probabilty weighted moments, L-moments, and so on. Expectation-maximization algorithm can also be utilised to find the maximum of the likelihood function since $\alpha$IW distribution has scale-mixture representation. However, in this study, the parameters of the $\alpha$IW distribution are estimated by using the ML, MPS, and LS methods for the complete sample case. Furthermore, only, the ML estimation method is considered for the progressively type-II censored sample case; see the appendix in the Supplementary Material. Therefore, estimation of the parameters of the $\alpha$IW distribution by using different estimation methods for complete and censored sample cases can be considered as future works.

### Supplementary Information

Supplementary Information.

## Data Availability

The data presented in this study are available in Section "Estimation"; see Tables 3 and 5.
