# Supplementary material for: A versatile model for lifetime of a component under stress
Source: Sci Rep. 2023 Nov 18;13:20202. doi: 10.1038/s41598-023-47313-3 (PMC10657370; doi:10.1038/s41598-023-47313-3)
Supplement: Supplementary file 1 — Supplementary Information. [file 41598_2023_47313_MOESM1_ESM.pdf]

Appendix

Appendix-A

Elements of the Hessian matrix are given below.

$$\frac{\partial \ln L}{\partial \alpha^2} = -\frac{2n}{\alpha^2} + \frac{n}{\beta^2}\Psi_1\left(\frac{\alpha}{\beta}\right) + \sum_{i=1}^{n} \frac{d}{d\alpha}\left[\frac{\frac{d}{d\alpha}G\left(t_i^{-\beta};\frac{\alpha}{\beta}+1,\sigma\right)}{G\left(t_i^{-\beta};\frac{\alpha}{\beta}+1,\sigma\right)}\right],$$

$$\frac{\partial \ln L}{\partial \alpha \partial \beta} = \frac{n}{\beta^2}\ln\sigma - \frac{n}{\beta^2}\left[\frac{\alpha}{\beta}\Psi_1\left(\frac{\alpha}{\beta}\right) + \Psi\left(\frac{\alpha}{\beta}\right)\right] + \sum_{i=1}^{n} \frac{d}{d\beta}\left[\frac{\frac{d}{d\alpha}G\left(t_i^{-\beta};\frac{\alpha}{\beta}+1,\sigma\right)}{G\left(t_i^{-\beta};\frac{\alpha}{\beta}+1,\sigma\right)}\right],$$

$$\frac{\partial \ln L}{\partial \alpha \partial \sigma} = -\frac{n}{\beta\sigma} + \sum_{i=1}^{n} \frac{d}{d\sigma}\left[\frac{\frac{d}{d\alpha}G\left(t_i^{-\beta};\frac{\alpha}{\beta}+1,\sigma\right)}{G\left(t_i^{-\beta};\frac{\alpha}{\beta}+1,\sigma\right)}\right],$$

$$\frac{\ln L}{\partial \beta^2} = -\frac{2n\alpha}{\beta^3}\ln\sigma + \frac{n}{\beta^2} + \frac{n\alpha^2}{\beta^4}\Psi_1\left(\frac{\alpha}{\beta}\right) + \sum_{i=1}^{n} \frac{d}{d\beta}\left[\frac{\frac{d}{d\beta}G\left(t_i^{-\beta};\frac{\alpha}{\beta}+1,\sigma\right)}{G\left(t_i^{-\beta};\frac{\alpha}{\beta}+1,\sigma\right)}\right],$$

$$\frac{\partial \ln L}{\partial \beta \partial \sigma} = \frac{n\alpha}{\beta^2\sigma} + \sum_{i=1}^{n} \frac{d}{d\sigma}\left[\frac{\frac{d}{d\beta}G\left(t_i^{-\beta};\frac{\alpha}{\beta}+1,\sigma\right)}{G\left(t_i^{-\beta};\frac{\alpha}{\beta}+1,\sigma\right)}\right],$$

and

$$\frac{\ln L}{\partial \sigma^2} = \frac{n\alpha}{\beta\sigma^2} + \sum_{i=1}^{n} \frac{d}{d\sigma^2}\left[\frac{\frac{d}{d\sigma}G\left(t_i^{-\beta};\frac{\alpha}{\beta}+1,\sigma\right)}{G\left(t_i^{-\beta};\frac{\alpha}{\beta}+1,\sigma\right)}\right].$$

Appendix-B

Let $\boldsymbol{T} = (T_{1:m:n}, T_{2:m:n}, \ldots, T_{m:m:n})$ with $T_{1:m:n} < T_{2:m:n} < \cdots < T_{m:m:n}$ be a progressively Type-II censored sample of size m under the censoring schema $\boldsymbol{R} = (R_1, R_2, \ldots, R_m)$. Then, the log-likelihood function based on progressively Type-II censored sample is

$$\begin{aligned}\ln L(\alpha,\beta,\sigma;T_{1:m:n},T_{2:m:n},\ldots,T_{m:m:n}) \simeq & 2m\ln\alpha - m\ln\beta - m\left(\frac{\alpha}{\beta}\right)\ln\sigma + m\ln\Gamma\left(\frac{\alpha}{\beta}\right) + (\alpha-1)\sum_{i=1}^{m}\ln t_{i:m:n} \\ & + \sum_{i=1}^{m}\ln\left[G\left(t_{i:m:n}^{-\beta};\frac{\alpha}{\beta}+1,\sigma\right)\right] + \sum_{i=1}^{m}R_i\ln\left[1-F\left(T_{i:m:n}\right)\right]\end{aligned}$$

where $F(\cdot)$ represents the cdf of the αIW distribution. After taking partial derivation the $\ln L$ function given in above with respect to the parameters of interest and setting them equal to 0, the following likelihood equations

$$\frac{\partial \ln L}{\partial \alpha} = \frac{2m}{\alpha} - \frac{m}{\beta}\ln\sigma + \frac{m}{\beta}\Psi\left(\frac{\alpha}{\beta}\right) + \sum_{i=1}^{m}\ln t_{i:m:n} + \sum_{i=1}^{m}\frac{\frac{d}{d\alpha}G\left(t_{i:m:n}^{-\beta};\frac{\alpha}{\beta}+1,\sigma\right)}{G\left(t_{i:m:n}^{-\beta};\frac{\alpha}{\beta}+1,\sigma\right)} + \sum_{i=1}^{m}\frac{\frac{d}{d\alpha}\left[1-F\left(t_{i:m:n}\right)\right]}{1-F\left(t_{i:m:n}\right)} = 0 \quad ,$$

$$\frac{\partial \ln L}{\partial \beta} = m\frac{\alpha}{\beta^2}\ln\sigma - \frac{m}{\beta} - m\frac{\alpha}{\beta^2}\Psi\left(\frac{\alpha}{\beta}\right) + \sum_{i=1}^{m}\frac{\frac{d}{d\beta}G\left(t_{i:m:n}^{-\beta};\frac{\alpha}{\beta}+1,\sigma\right)}{G\left(t_{i:m:n}^{-\beta};\frac{\alpha}{\beta}+1,\sigma\right)} + \sum_{i=1}^{m}\frac{\frac{d}{d\beta}\left[1-F\left(t_{i:m:n}\right)\right]}{1-F\left(t_{i:m:n}\right)} = 0 \quad ,$$

and

$$\frac{\partial \ln L}{\partial \sigma} = -m\frac{\alpha}{\beta\sigma} + \sum_{i=1}^{m}\frac{\frac{d}{d\sigma}G\left(t_{i:m:n}^{-\beta};\frac{\alpha}{\beta}+1,\sigma\right)}{G\left(t_{i:m:n}^{-\beta};\frac{\alpha}{\beta}+1,\sigma\right)} + \sum_{i=1}^{m}\frac{\frac{d}{d\sigma}\left[1-F\left(t_{i:m:n}\right)\right]}{1-F\left(t_{i:m:n}\right)} = 0$$

are obtianed. As similar to complete sample case, simulatenous solutions of the likelihood equation given above give the ML estimates of the parameters α, β, and σ of the αIW distribution under progressively Type-II censored sample.
